# Chromosome-Scale Assembly and Annotation of the Macadamia Genome (*Macadamia integrifolia* HAES 741)

**DOI:** 10.1534/g3.120.401326

**Published:** 2020-08-03

**Authors:** Catherine J. Nock, Abdul Baten, Ramil Mauleon, Kirsty S. Langdon, Bruce Topp, Craig Hardner, Agnelo Furtado, Robert J. Henry, Graham J. King

**Affiliations:** * Southern Cross Plant Science, Southern Cross University, Lismore NSW 2480, Australia; † AgResearch NZ, Grasslands Research Centre, Palmerston North 4442, New Zealand; ‡ Queensland Alliance for Agriculture and Food Innovation, University of Queensland, St Lucia QLD 4069, Australia

**Keywords:** Proteaceae, genome, pseudo-chromosome, transcriptome, nut crop

## Abstract

*Macadamia integrifolia* is a representative of the large basal eudicot family Proteaceae and the main progenitor species of the Australian native nut crop macadamia. Since its commercialisation in Hawaii fewer than 100 years ago, global production has expanded rapidly. However, genomic resources are limited in comparison to other horticultural crops. The first draft assembly of *M. integrifolia* had good coverage of the functional gene space but its high fragmentation has restricted its use in comparative genomics and association studies. Here we have generated an improved assembly of cultivar HAES 741 (4,094 scaffolds, 745 Mb, N50 413 kb) using a combination of Illumina paired and PacBio long read sequences. Scaffolds were anchored to 14 pseudo-chromosomes using seven genetic linkage maps. This assembly has improved contiguity and coverage, with >120 Gb of additional sequence. Following annotation, 34,274 protein-coding genes were predicted, representing 90% of the expected gene content. Our results indicate that the macadamia genome is repetitive and heterozygous. The total repeat content was 55% and genome-wide heterozygosity, estimated by read mapping, was 0.98% or an average of one SNP per 102 bp. This is the first chromosome-scale genome assembly for macadamia and the Proteaceae. It is expected to be a valuable resource for breeding, gene discovery, conservation and evolutionary genomics.

The genomes of most crop species have now been sequenced and their availability is transforming breeding and agricultural productivity (Bevan *et al.* 2017, Chen *et al.* 2019). Macadamia is the first Australian native plant to become a global food crop. In 2018/2019, world production was valued at US$1.1 billion (59,307 MT/year, kernel basis), reflecting the most rapid increase in production of any nut crop over the past 10 years (International Nut and Dried Fruit Council, http://www.nutfruit.org). As a member of the Gondwanan family Proteaceae (83 genera, 1660 species) (Christenhusz and Byng 2016), *Macadamia* (F.Muell.) is a basal eudicot and is phylogenetically divergent from other tree crops (Nock *et al.* 2014)_._ *M. integrifolia*, the main species used in cultivation (Hardner 2016), is a mid-storey tree endemic to the lowland rainforests of subtropical Australia (Powell *et al.* 2014) (Figure 1). Macadamia was commercialised as a nut crop from the 1920s in Hawaii and cultivated varieties are closely related to their wild ancestors in Australia (Nock *et al.* 2019).

**Figure 1:**
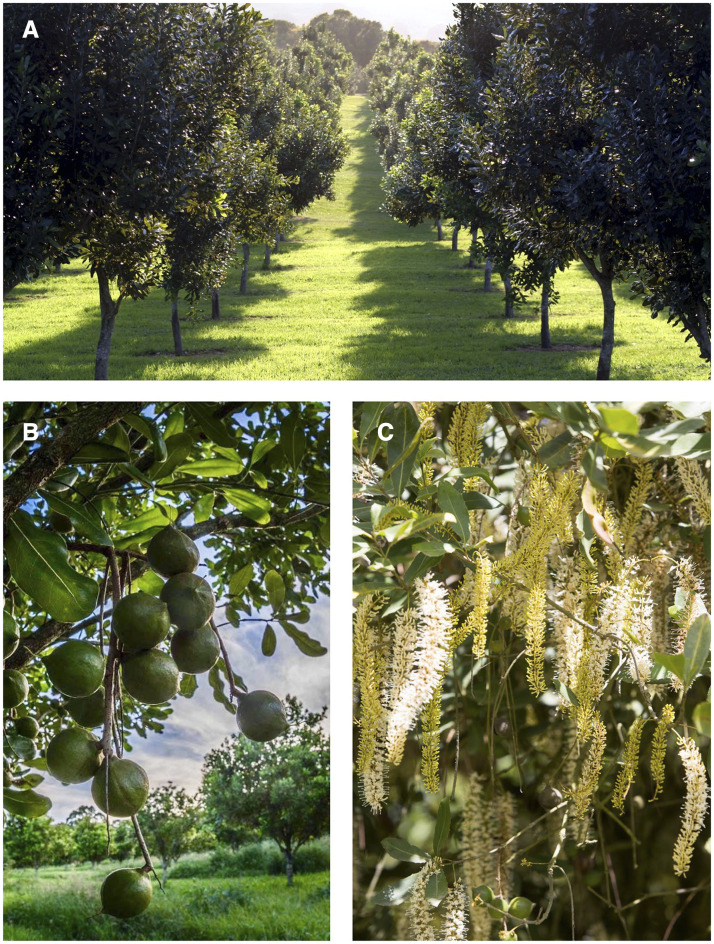
*Macadamia integrifolia* (a) orchard (b) nut in husk (c) racemes.

Despite the rapid expansion in production over the past 50 years and commercial cultivation in 18 countries, breeding is restricted by a paucity of information on the genes underlying important crop traits (Topp *et al.* 2019). There are few genomic resources for either macadamia or within the Proteaceae. Transcriptomic data are available for *Macadamia* (Chabika *et al.* 2020) and other Proteaceae genera including *Banksia* (Lim *et al.* 2017), *Grevillea* (Damerval *et al.* 2019)_,_ *Protea* (Akman *et al.* 2016) and *Gevuina* (Ostria-Gallardo *et al.* 2016)_,_ and have been utilized to help to understand the evolution of floral architecture (Damerval *et al.* 2019) and variation in locally adaptive traits (Akman *et al.* 2016). Recent genome wide association studies in macadamia have identified markers associated with commercially important traits (O’Connor *et al.* 2020) but their location and context in the genome is unknown. An earlier highly fragmented (193,493 scaffolds, N50 4,745) draft genome assembly of the widely grown *M. integrifolia* cultivar HAES 741 was constructed from Illumina short read sequence data (Nock *et al.* 2016)_._

Here we report on the first annotated and anchored genome assembly for macadamia and the Proteaceae. Long read PacBio and paired-end Illumina sequence data were generated and 187 Gb of data were used to construct an improved HAES 741 assembly (Table 1). Assembled sequence scaffolds from the new hybrid *de novo* assembly (745 Mb, 4094 scaffolds, N50 413 kb) were then anchored and oriented to 14 pseudo-chromosomes using methods to maximize colinearity across seven genetic linkage maps (Langdon *et al.* 2020). A contiguous genome sequence for macadamia is expected to facilitate the identification of candidate genes and support marker assisted selection to accelerate the development of new improved varieties.

**Table 1:** Data files and library information for *Macadamia integrifolia* genome sequencing. *Data deposited for draft assembly v1.1

Sequencing Platform	Library	Insert size (bp)	Read length (bp)	Sequence reads (Million)	Sequence bases (Gb)	Accession
Genomic data						
Illumina	Pair end	200	2 x 125	228.8	57.2	SRR10896963
	Pair end	350	2 x 125	119.0	29.8	SRR10896962
	Pair end	550	2 x 125	107.6	26.9	SRR10896961
	Pair end	480	2 x 150	58.7	17.7	ERX1468522-23*
	Pair end	700	2 x 150	28.7	8.7	ERX1468524*
	Mate pair	8000	2 x 100	200.3	40.5	ERX1468525*
PacBio	NA		9769	1.0	6.4	10896960
**Total gDNA**				**744.1**	**187.2**	
Transcriptomic data						
Illumina	young leaf	300	2 x 100	77.9	15.6	SRR10897159
	shoot	300	2 x 100	71.6	14.3	SRR10897158
	flower	300	2 x 100	83.3	16.7	SRR10897157
**Total RNA-seq**				**232.8**	**46.6**	

## Materials and Methods

### Sample collection and extraction

Leaf, shoot and flower tissue were collected from a single *M. integrifolia* cultivar HAES 741 individual located in the M2 Regional Variety Trial plot, Clunes, New South Wales, Australia (28°43.843’S; 153°23.702’E). An herbarium specimen was submitted to the Southern Cross Plant Science Herbarium [accession PHARM-13-0813]. On collection, samples were snap frozen in liquid nitrogen or placed on dry ice and stored at -80° prior to extraction. Previously described methods for and DNA/RNA extraction for Illumina (Nock *et al.* 2016) and PacBio (Furtado 2014) sequencing were followed.

### Library preparation and sequencing

In addition to the original 480 bp and 700 bp insert and 8 kb mate pair libraries (51.6 Gb total), new libraries with 200, 350 and 550 bp insert sizes were prepared with TruSeq DNA PCR-free kits and sequenced with Illumina HiSeq 2500 producing 57.0, 29.8 and 29.6 Gb paired-end sequence data. A PacBio library (20-Kb) was prepared and sequenced across 10 SMRT cells on a PacBio RSII system (P6-C4 chemistry) generating 6.44 Gb data. Transcriptome sequencing of leaf, shoot and flower tissue followed previously described methods (Nock *et al.* 2016) and generated 44.6 Gb paired-end RNA-seq data in total (Table 1).

### De novo assembly and anchoring

Illumina raw read data were initially assessed for quality using FastQC (Andrews 2010)· Low quality bases (Q < 20), adapters and chloroplast reads were removed from Illumina data using BBMaps (Bushnell 2020). PacBio reads were error-corrected using the Long Read DBG Error Correction method (LoRDEC) with over 170 × post-QC Illumina data (Salmela and Rivals 2014). The performance of hybrid error correction methods including LoRDEC is optimized with increasing coverage of more accurate short, rather than long read, sequence data since each long read is corrected independently (Fu *et al.* 2019). Hybrid *de novo* assembly, scaffolding and gap closing was performed using MaSuRCA (Zimin *et al.* 2013). Illumina paired-end and mate-pair reads were extended into super-reads of variable lengths, and combined with PacBio reads to generate the assembly. The MaSURCA output was further scaffolded in two rounds, first using SSPace (Boetzer *et al.* 2011) scaffolder with the Illumina mate pair reads, and second, using L_RNA_Scaffolder (Xue *et al.* 2013) with the long transcripts generated using a Trinity (Grabherr *et al.* 2011) pipeline and RNA-Seq reads. Scaffolds were anchored and oriented with 100 bp gap size between scaffolds using ALLMAPS (Tang *et al.* 2015) with 4,266 ordered sequence-based markers across seven genetic linkage maps generated from four mapping populations with HAES 741 parentage (Langdon *et al.* 2020).

### Genome size and heterozygosity

For genome size and heterozygosity estimation, k-mer frequency was determined from cleaned short and long read data for k ranging from 15 to 33 at a step of two in Jellyfish (Marcais and Kingsford 2011). Genome size was estimated at the optimal k-mer of 25, with short read data only using findGSE because it is an optimal method for plants (Sun *et al.* 2018). Genome-wide heterozygosity using reads data were estimated with GenomeScope (Vurture *et al.* 2017) from the k-mer 25 histogram computed using Jellyfish. In addition, genome-based heterozygosity was determined by mapping post-QC short read data to the assembly using minimap2 (Li 2018) and heterozygous sites identified using GATK best practices (Van der Auwera *et al.* 2013) for SNP discovery.

### Genome annotation and gene prediction

Repetitive elements were first identified in the final assembly by modeling repeats using RepeatModeler, and then quantified using RepeatMasker (Tarailo-Graovac and Chen 2009). Transcriptome assembly was performed using the Trinity pipeline (Grabherr *et al.* 2011). Following repeat masking, the final assembly was annotated using the MAKER gene model prediction pipeline (Campbell *et al.* 2014). Sources of evidence for gene prediction included the Trinity assembled transcripts and protein sequences of the taxonomically closest available genome sequence of the sacred lotus *Nelumbo nucifera*, and the model plant *Arabidopsis thaliana*.

### Comparative analysis of orthologous eudicot genes

Orthologous gene clusters were identified and compared using OrthoVenn2 (Zu *et al.* 2019) and protein sequences from *M. integrifolia*, *Nelumbo nucifera*, *Arabidopsis thaliana*, *Prunus persica* (peach), *Eucalpytus grandis* and *Coffea canephora* (coffee) genomes. Pairwise sequence similarities were determined applying a BLASTP E-value cut-off of 1E-05 and an inflation value of 1.5 for OrthoMCL Markov clustering. Macadamia specific gene clusters were tested for GO enrichment using OrthoVenn2.

### Quality assessment

The completeness of the genome assembly was evaluated by BUSCO (benchmarking universal single copy orthologs) (Simão *et al.* 2015) using 956 plant BUSCOs. To further assess the accuracy of the *M. integrifolia* genome assembly, short read and corrected long read data were independently mapped to the pseudo-genome using Minimap2 (Li 2018) and the number of mapped reads were computed using paftools.js. The final step of the LoRDEC error correction method applied involves trimming and splitting PacBio reads by extracting runs of solid bases as separate reads (Salmela and Rivals 2014). To test for chimerism, Pacbio reads > 100 bp in length we re-aligned to the assembly using cutoffs of 1/2 and 1/3 read length.

### Data availability

The macadamia Whole Genome Shotgun project has been deposited at DDBJ/ENA/GenBank under the accession JAAEEG000000000. The version described in this paper is JAAEEG010000000. Datasets generated in this study have been deposited at NCBI under BioProject number PRJNA593881, https://www.ncbi.nlm.nih.gov/bioproject/PRJNA593881 Raw genomic DNA and RNA-seq read files have been deposited in the NCBI Sequence Read Archive (Table 1). Datasets including protein, transcript and gff3 annotation files, and raw trinity transcriptome assembly have been deposited in the Southern Cross University data repository, https://doi.org/10.25918/5e320fd1e5f06.

## Results and Discussion

### Genome assembly

**Figure 2:**
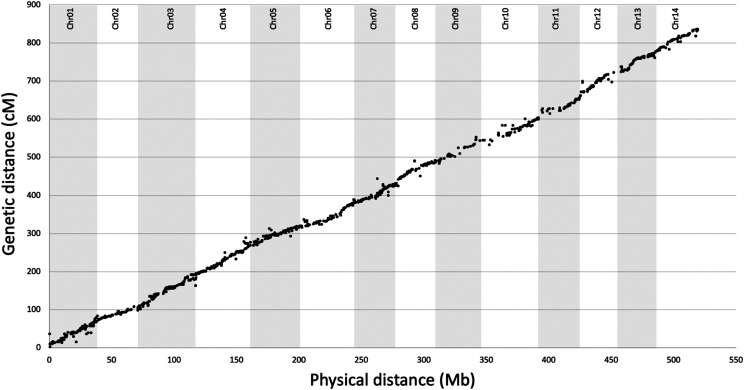
Genetic length (centimorgans, cM) *vs.* physical length (megabases, Mb) plotted for the *Macadamia integrifolia* cv. HAES 741 genome.

Hybrid *de novo* assembly using 180.8 Gb Illumina short read and 6.44 Gb PacBio long read data produced a 744.6 Mb genome assembly (v2) with 180 × coverage of the genome and a scaffold N50 of 413 kb (Table 1). In comparison to the v1.1 assembly of the same cultivar, this v2 assembly represents an ∼68 fold increase in contiguity and includes 120.3 Mb of new sequence (Table 2). Scaffolds were anchored and oriented to 14 pseudo-chromosomes using ALLMAPs to maximize the collinearity of ordered sequence-based markers across seven genetic linkage maps generated from mapping populations with HAES 741 parentage. Of 1,465 anchored scaffolds, 474 were N50 scaffolds and 856 containing more than one marker were oriented (Langdon *et al.* 2020). This anchored 69.7% of the assembly to 14 pseudo-chromosomal sequences ranging in size from 29.2 to 47.0 Mb (Table 3). Mapped markers were evenly distributed across the 14 pseudo-chromosomes with the exception of some gapped regions particularly on Chr10 and Chr12 (Figure 2).

**Table 2:** Comparison of the new *Macadamia integrifolia* cv. HAES 741 genome assembly with the previously published draft assembly. Coverage is based on k-mer estimated genome size of 895.7 Mb. Gaps are ambiguous base calls

	Data		Scaffold			Assembly			Annotation		
	Post-QC Gb	Genome Coverage X	Number	N50 kb	Longest Mb	Assembled Genome Mb	Gaps Mb	Genome Coverage %	Repeats	Gene Models	Expected Gene content
v1.1	51.6	58	193,493	4.7	0.64	518.49	70	58%	37%	35,337	77.4%
v2	161.5	180	4094	413.4	2.19	744.64	11	83%	55%	34,274	90.2**%**

**Table 3:** Summary of the assembled chromosomes of macadamia

Chromosome	Length bp	scaffolds	genes
Chr01	36,399,236	126	1543
Chr02	44,010,915	120	2214
Chr03	37,831,390	112	2039
Chr04	37,507,012	91	2133
Chr05	46,991,412	110	2334
Chr06	40,842,004	101	1941
Chr07	36,789,931	112	1940
Chr08	34,869,527	94	1975
Chr09	42,247,492	109	2019
Chr10	34,167,173	87	1701
Chr11	33,967,801	107	1488
Chr12	31,654,987	87	1523
Chr13	29,219,948	100	1398
Chr14	32,845,142	109	1531
**Anchored**	**519,343,970**	**1465**	**25,779**
Unplaced	225,433,603	2629	8495
**Total**	**744,777,573**	**4094**	34,274

The quality and completeness of the assembly, assessed using BUSCO, indicates that the macadamia assembly contains 90.2% of the expected single copy gene content. In comparison to the previous assembly of the same cultivar, 16.5% compared to 39.2% of the plant BUSCOs searched were fragmented. The accuracy of the assembly was assessed by read mapping with 99% of short reads (98.97–99.15% per library) and 93.5% of corrected PacBio reads tagged as primary alignments by Minimap2 aligned reliably to the assembly. No Pacbio reads aligned to two different scaffolds at a cutoff of 1/2 read length. At 1/3 read length, 130 reads (mean length 7833 bp representing < 0.14% of the assembly) aligned to two scaffolds and no reads aligned to more than two scaffolds.

### Genome size, heterozygosity and repetitive content

Genome size estimation of 896 Mb was 1.37 times larger than the only previous estimate for *M. integrifolia* of 652 Mb (600-700 Mb) that was based on 51.6 Gb Illumina short read data (Nock *et al.* 2016). The new k-mer based estimate is considered to be more accurate due to improved read coverage from an additional 114 Gb of high-quality Illumina data. Based on the revised genome size estimate, the v2 assembly covers 83% of the genome.

**Figure 3:**
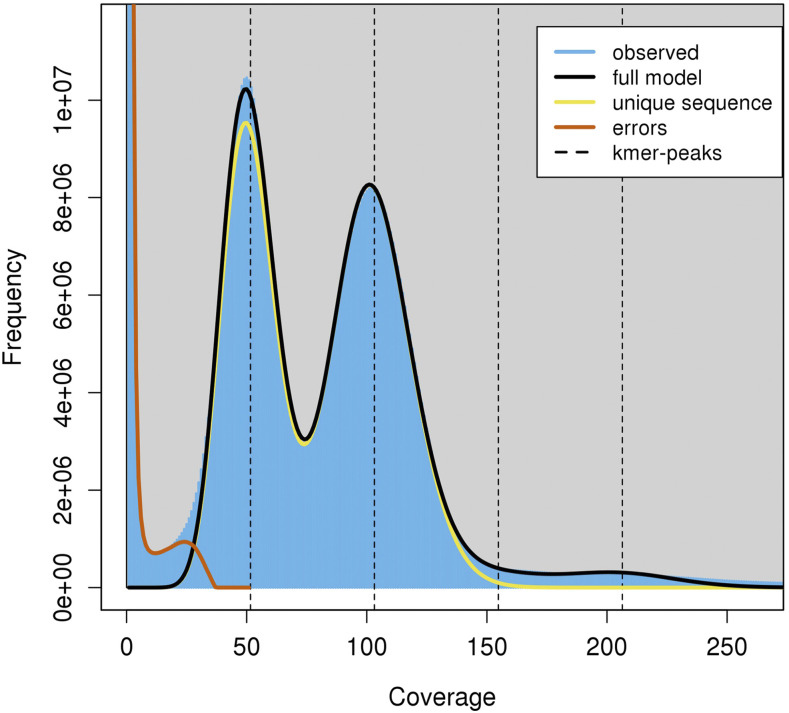
The 25-mer distribution for estimation of genome heterozygosity and size. Peaks at approximately 50, 100 and 200 represent heterozygous, homozygous and repeated k-mers respectively.

**Figure 4:**
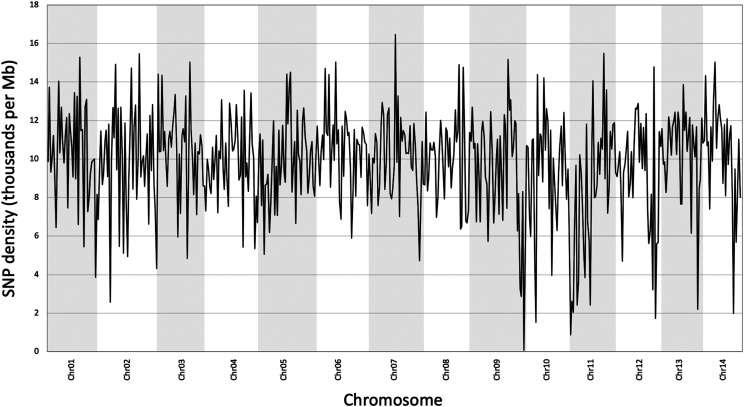
Genome wide SNP density. Thousands of SNPs per 1 Mb window, shown across each chromosome.

Average genome heterozygosity, determined using short read data from the k-mer 25 Jellyfish histogram, was 1.36% (Figure 3). Genome-wide heterozygosity was slightly lower at 0.98% for the assembly-based SNP analysis method (Li 2018, Van der Auwera *et al.* 2013). In total, 7,309,539 heterozygous sites were identified in the HAES 741 genome assembly representing one SNP per 102 bp and consistent with reports that macadamia is highly heterozygous and predominantly outcrossing (Topp *et al.* 2019). Heterozygous sites were unevenly distributed across chromosomes. On average, there were 367,467 SNPs per chromosome and SNP density ranged from 66 to 16,453 SNPs in one Mb windows of Chr07 and Chr10 respectively (Figure 4).

**Figure 5:**
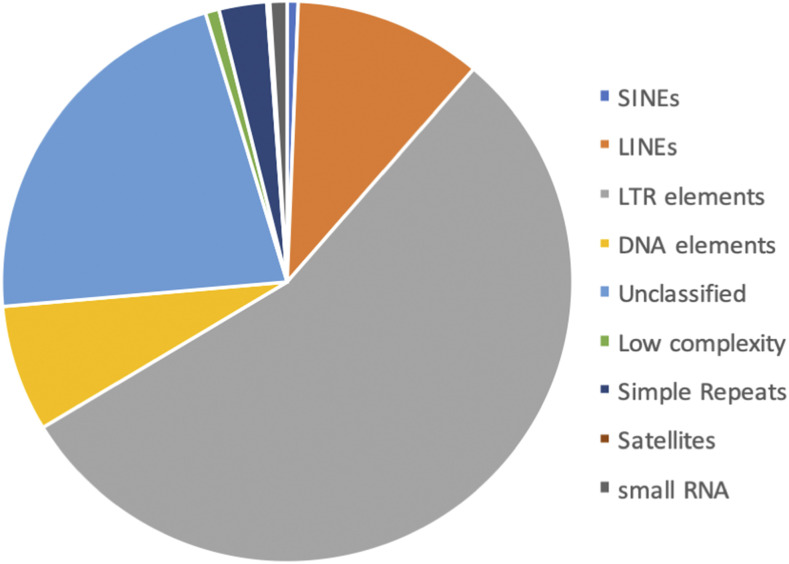
Interspersed repeats and low complexity elements representing 55.1% of the macadamia genome assembly.

Interspersed repeats and low complexity elements accounted for 410.5Mb (55.1%) of the assembly. As reported for many other plant genomes, long terminal repeat (LTR) retrotransposons were the most abundant repeat type (30.5%). Small RNAs including pseudogenes and active small RNA genes including tRNA and snRNA accounted for 0.54% of the assembly (Figure 5).

### Annotation and comparative analysis of orthologous genes

**Figure 6:**
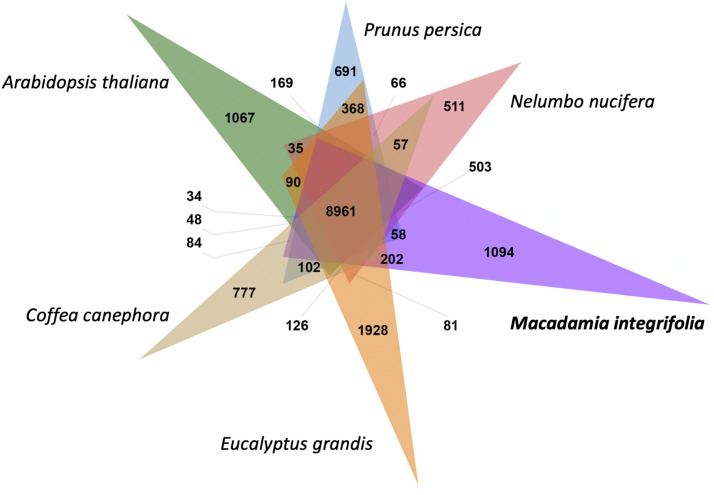
Venn diagram of orthologous gene clusters for six eudicot species including the basal eudicots *M. integrifolia* and *N. nucifera* (Proteales) and core eudicots *P. persica* (Rosales), *A. thaliana* (Brassicales), *C. canephora* (Gentianales) and *E. grandis* (Myrtales).

Annotation of the final assembly, identified 34,274 high-confidence gene models. Of these, 25,779 (75.2%) were anchored to pseudo-chromosomes and 8,495 were located on unanchored scaffolds (Table 3). Comparative analysis of the macadamia gene models with the proteins of *N. nucifera*, *A. thaliana*, *P. persica*, *E. grandis* and *C. canephora* identified 8,961 orthologous clusters of which 2,051 contained a single gene from each of the six eudicot species. *M. integrifolia* and *N. nucifera* both belong to the basal eudicot order Proteales. These species and contained similar numbers of clusters with 13,491 and 13,321 respectively and shared the largest number of clusters (503) of any species pair (Figure 6). Tests for gene ontology (GO) enrichment of 1,094 macadamia specific clusters identified five significant terms including those associated with the defense response (GO:0006952, *P* = 4.8E-05) and fruit ripening (GO:0009835, *P* = 5.9E-04).

## Conclusion

Here we present the first chromosome-scale genome assembly for the nut crop macadamia and the large Gondwanan plant family Proteaceae. This provides a platform for unraveling the genetics of macadamia and is expected to underpin future breeding, and comparative and horticultural genomics research.

### Code availability

Versions and parameters of the tools implemented in data analysis are provided below:

BBMap version 36.62: ktrim = r k = 23 mink = 11 hdist = 1 tpe tbo maxns = 1 minlen = 50 maq = 8 qtrim = rl trimq = 20

LoRDEC version 0.4: lordec-correct -2 input_for_read_correction.fastq -k 19 -S out.stat.txt -s 3 -T 12 -i PacBio_filtered_reads.fasta -o out.pacbio.corrected.fasta

MaSuRCA version 3.2.1. Default parameters except: *NUM_THREADS = 16*, *JF_SIZE = 20000000000* (jellyfish hash size)

L_RNA_scaffolder: blat -t = dna -q = dna scaffolds_gapClosed_min1000.fa Trinity.fasta transcript.*vs.*macaAssembly.psl -noHead -out = psl

ALLMAPS version 0.7.7: Default parameters

Jellyfish version 2.0: jellyfish count -t 14 -C -m 27 -s 8G -o 27mer_maca_illumOnly_out <all Illumina-only WGS fastqs > jellyfish histo -t14 27mer_maca_illumOnly_out> 27mer_maca_illumOnly_out.histo

findGSE: Default parameters

GenomeScope version 2.2.6:kmer length 27, read length 125bp, Max kmer coverage 1000

Trinity, version 2.0.3: Trinity–seqType fq–max_memory 100G –left reads_S_R1_clean.fq,reads_F2_R1_clean.fq,reads_YL_R1_clean.fq–right reads_S_R2_clean.fq,reads_F2_R2_clean.fq,reads_YL_R2_clean.fq–CPU 8

RepeatModeler version 2.0.1. Default parameters

RepeatMasker version 4.0.9. Default parameters

MAKER, version 2.31.10. Default parameters except: Gene prediction methods Augustus and SNAP (trained with previously generated macadamia gene models); AED score = 0.40; Minimum protein length: = 50 amino acids

BUSCO version 3.0.2: busco -i proteins.fasta -l viridiplantae_odb10 -m proteins -o output_name

Minimap2 version 2.17. Default parameters (paftool.js bundled with Minimap2): minimap2 -ax sr ref_assemblyfasta fastq1stReadPair fastq2ndReadPair > ref_readPairs_aln.paf; k8 paftools.js stat ref_readPairs_aln.paf > alignment_mapstats

GATK HaplotypeCaller version 4.1.4.1: gatk–java-options “-Xmx8g” HaplotypeCaller -R refGenome.fasta -I input.bam -O output.g.v*cf*.gz -ERC GVCF -heterozygosity 0.01

GATK GenotypeGVCFs version 4.1.4.1: gatk–java-options “-Xmx4g” GenotypeGVCFs -R refGenome.fasta -V input.g.v*cf*.gz -O output.v*cf*.gz–heterozygosity 0.01

GATK VariantsToTable version 4.1.4.1: gatk-4.1.4.1/gatk–java-options “-Xmx8g” VariantsToTable -V output.v*cf*.gz -F CHROM -F POS -F TYPE -F REF -F ALT -F HET -GF GT -GF AD -O output_genoGV*CF*.table.txt.
